# Trained Immunity-Based Vaccine in B Cell Hematological Malignancies With Recurrent Infections: A New Therapeutic Approach

**DOI:** 10.3389/fimmu.2020.611566

**Published:** 2021-02-12

**Authors:** Juliana Ochoa-Grullón, Celina Benavente Cuesta, Ataúlfo González Fernández, Gustavo Cordero Torres, Cristina Pérez López, Ascensión Peña Cortijo, Laura Conejero Hall, Marta Mateo Morales, Antonia Rodríguez de la Peña, Carmen M. Díez-Rivero, Edgard Rodríguez de Frías, Kissy Guevara-Hoyer, Miguel Fernández-Arquero, Silvia Sánchez-Ramón

**Affiliations:** ^1^ Department of Clinical Immunology, IML and IdISSC, Hospital Clínico San Carlos, Madrid, Spain; ^2^ Department of Immunology, Ophthalmology and ENT, School of Medicine, Complutense University, Madrid, Spain; ^3^ Department of Hematology, IML, Hospital Clínico San Carlos, Madrid, Spain; ^4^ R+D Department, Inmunotek S.L., Alcalá de Henares, Madrid, Spain

**Keywords:** hematological malignancies, IgA, recurrent respiratory tract infections, trained immunity-based vaccines, MV130, prophylaxis

## Abstract

Infectious complications are a major cause of morbidity and mortality in B-cell hematological malignancies (HM). Prophylaxis for recurrent infections in HM patients with antibody deficiency consists of first-line antibiotics and when unsuccessful, gammaglobulin replacement therapy (IgRT). Recent knowledge of trained immunity-based vaccines (TIbV), such as the sublingual polybacterial formulation MV130, has shown a promising strategy in the management of patients with recurrent infections. We sought to determine the clinical benefit of MV130 in a cohort of HM patients with recurrent respiratory tract infections (RRTIs) who underwent immunization with MV130 for 3 months. Clinical information included the frequency of infections, antibiotic use, number of visits to the GP and hospitalizations previous and after MV130 immunotherapy. Improvement on infection rate was classified as: clear (>60% reduction of infection), partial (26%–60%) and low (≤25%) improvement. Fifteen HM patients (aged 42 to 80 years; nine females) were included in the study. All patients reduced their infection rate. Analysis of paired data revealed that the median (range, min - max) of respiratory infectious rate significantly decreased from 4.0 (8.0–3.0) to 2.0 (4.0–0.0) (*p*<0.001) at 12 months of MV130. A clear clinical improvement was observed in 53% (n = 8) of patients, partial improvement in 40% (n = 6) and low improvement in 7% (n = 1). These data correlated with a decrease on antibiotic consumption from 3.0 (8.0–1.0) to 1.0 (2.0–0.0) (*p* = 0.002) during 12 months after initiation of treatment with MV130. The number of infectious-related GP or emergency room visits declined from 4.0 (8.0–2.0) to 2.0 (3.0–0.0) (*p*<0.001), in parallel with a reduction in hospital admissions due to infections (*p* = 0.032). Regarding safety, no adverse events were observed. On the other hand, immunological assessment of serum IgA and IgG levels demonstrated an increase in specific antibodies to MV130-contained bacteria following MV130 immunotherapy. In conclusion, MV130 may add clinical benefit reducing the rate of infections and enhancing humoral immune responses in these vulnerable patients.

## Introduction

B cell hematological malignancies (HM) are a diverse group of hematological diseases where both the disease and the use of B cell therapies contribute to secondary immunodeficiency (SID) (1). HM patients are particularly prone to recurrent and severe infections (2, 3), which may result in prolonged hospital admissions, increases in healthcare costs and second-line drugs, treatment failures and subsequent, patient’s fragility and reduced quality of life (4, 5). Antibiotic prophylaxis is the mainstay of management in patients with recurrent infections (6, 7). However, the advent of multidrug resistance among pathogenic bacteria poses a serious global threat of growing concern (8–10). Moreover, the use of antibiotics in recurrent infections might have: i) deleterious effects on the microbiota, such as an enhancement of pathogen invasion; ii) potential adverse effects, including nephrotoxicity and hepatotoxicity; and, iii) limitations for treating diverse microorganisms. In addition, in the particular case of respiratory tract infections, influenza and other viral pathogens are the most common causes of acute respiratory infections, predisposing patients to secondary bacterial infections often leading to more severe clinical outcomes. Therefore, the development of alternative strategies or adjuvant approaches are a priority for preventive purposes (11).

Within the immunosuppressive environment of many HM, it has been suggested that innate immune cells may be a valuable target for infection prevention by inducing the so-called “trained immunity” (12). Trained immunity is described as a long-term (up to 1-year) boosting of innate immune responses by certain pathogens or pathogen-associated molecular patterns, including specific vaccines, which leads to heterologous protection against infection through epigenetic, transcriptional and functional reprograming of innate immune cells (13). Therefore, trained immunity-based vaccines (TIbV) provide the potential for identifying novel therapeutic targets that protect from infections and reverse immunotolerant states (14). TIbV enhance and induce long lasting epigenetic changes in innate immune cells, enabling quicker clearance of microbial infections (15). Thus, we foresee that sublingual mucosal TIbV could represent a good adjuvant or alternative to antibiotics as a prophylactic intervention.

Different types of mucosal bacterial vaccines, some of them included in the TIbV category, have gained attention for the treatment of recurrent respiratory tract infections (RRTIs) over the last decades (16–20). In this regard, MV130 is a sublingual bacterial preparation formulated with heat-inactivated whole-cell bacteria that has shown to provide clinical benefit in patients with RRTIs of different etiology, including bacteria and viruses (19, 21). In addition, it was successfully used in a clinical trial in children with recurrent wheezing, a condition triggered in most cases by viral infections (22). Its immunomodulatory mechanism of action has been further studied *in vitro* and *in vivo* (23, 24). MV130 is a mucosal TIbV used to prevent infections in patients with recurrent infections without known immunodeficiency, but also may have that role in immunocompromised patients (25, 26). Mucosal TIbVs act directly on specific tissues where pathogens initiate or spread infections, decreasing the number of reinfections and also inducing tolerance (23, 27). In addition, by innate immune training, infection incidence might be reduced in the high-risk setting of cancer therapy.

The aim of this study was to evaluate for the first time the clinical benefit of mucosal immunotherapy with MV130 in HM patients with recurrent infections in routine clinical practice. The results indicate that MV130 is a safe and promising strategy for treating recurrent respiratory infections in this highly vulnerable population, leading to reduction in the rate of RRTIs, antibiotic consumption and other healthcare resources, together with an increase in specific IgA and IgG antibodies to MV130-containing bacteria indicating its immunogenicity in these patients.

## Methods

### Patients and Study Design

**Figure 1:**
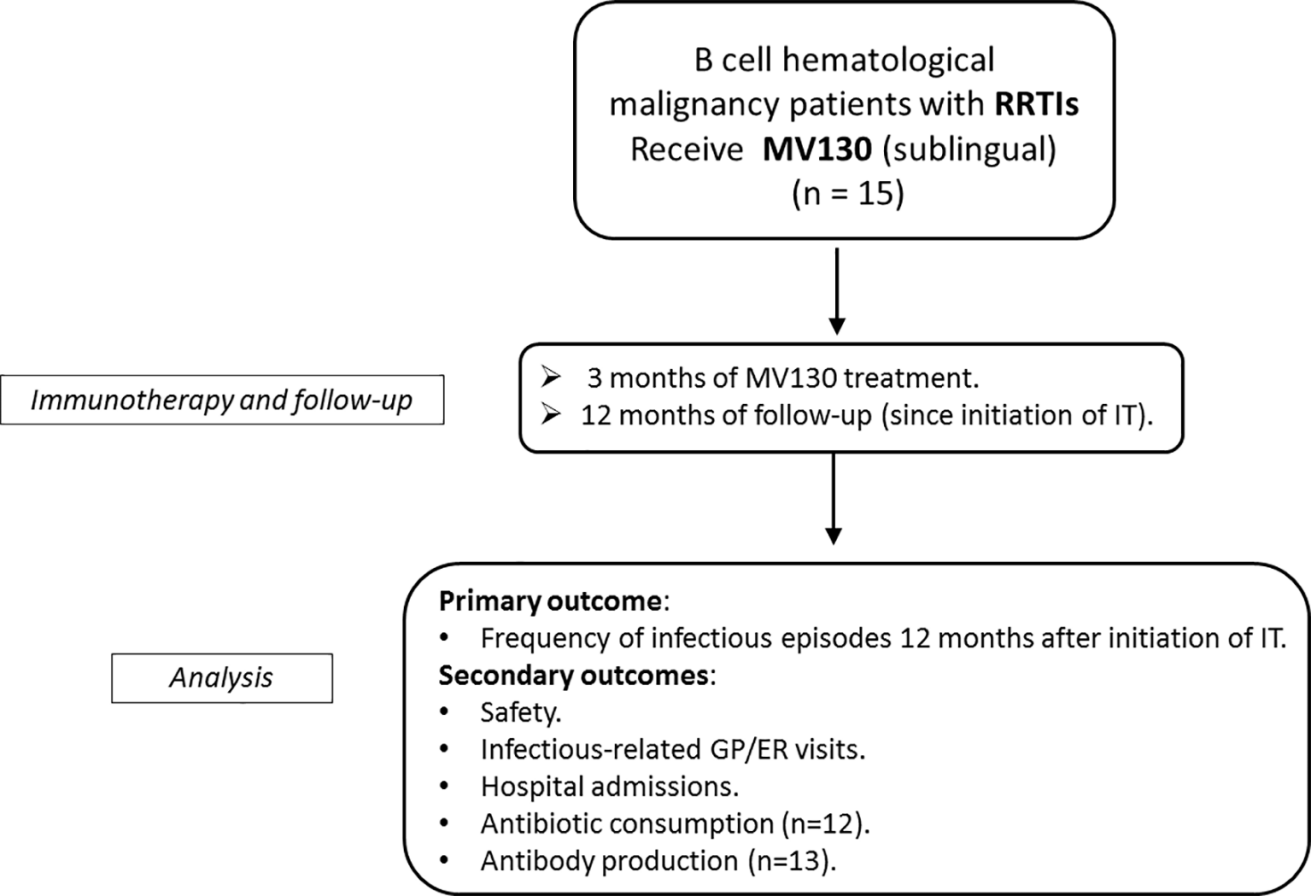
Flow chart of the study including enrolment, medical intervention, therapy and follow-up, and analysis. RRTIs, recurrent respiratory tract infection; IT, immunotherapy; GP, general practitioner; ER, emergency room.

We carried out a single-center retrospective observational pilot study. Between 2015 and 2017, a cohort of 15 patients diagnosed with HM based on the WHO Classification of Tumours of Hematopoietic and Lymphoid Tissues (28) and RRTI were consecutively referred from the Hematology Department to the Clinical Immunology Department for evaluation of suspected immunodeficiency. Patients were classified according to the following criteria and based on infectious data of the previous 12 months before TIbV initiation: i) recurrent upper respiratory tract infection (URTI): defined as ≥3 episodes of rhinitis, sinusitis, otitis, pharyngitis or tonsilitis; ii) recurrent lower respiratory tract infection (LRTI) defined as ≥1 episode of acute bronchitis, pneumonia or community-acquired pneumonia, acute exacerbation of chronic obstructive pulmonary disease (COPD) or bronchiectasis. All patients were put on sublingual bacterial immunotherapy (MV130) (n = 15) (**Figure 1**) and were monitored closely for their safety and clinical evaluation at each study visit (0, 3-, 6- and 12-months), as per routine clinical practice. Clinical information includes the frequency of infections, antibiotic use, number of visits to the GP and infections-related hospitalizations previous and after MV130 immunotherapy. Approval for the study was obtained from the Hospital Clínico San Carlos, Madrid (Spain) institutional research Ethics Committee (19/219-E). The study was performed in accordance with Spanish national guidelines. Data were collected conforming medical records from out-patient clinics.

### Bacterial Immunotherapy

MV130 (Bactek^®^, Inmunotek, Spain) is a suspension of heat-inactivated whole cell bacteria (90% Gram +ve [V101 *Staphylococcus epidermidis*, V102 *S. aureus*, V104 *Streptococcus pneumoniae*] and 10% Gram -ve [V103 *Haemophilus influenzae*, V105 *Moraxella catarrhalis*, V113 *Klebsiella pneumoniae*]) at 300 Formazin Turbidity Units (FTU)/ml (~ 10^9^ bacteria/ml). The treatment was administered perlingually by spraying under the tongue two puffs of 100 μl each, daily for 3 months. To check the correct administration of the medication, the first dose was administered in the hospital; the following doses were administered at home. It should be avoided concomitant intake of food or beverage, but it can be applied any time of the day. The bacterial suspension is maintained under the tongue for at least 1–2 min.

### Outcomes

The frequency of infectious episodes 12-months prior and 12-months after MV130 treatment was considered the primary outcome of the study. Patients were classified based on their improvement on infection rate in three groups: clear (>60% reduction of infections), partial (26%–60%) and low (≤25%) improvement.

Secondary outcomes included: safety assessment, infectious-related unscheduled general practitioner (GP) or Emergency room visits, hospital admission due to infections, number of cycles of antibiotic prescription and specific IgA and IgG sera titers before and after immunotherapy with MV130 (**Figure 1**).

#### Serum IgA and IgG Measurement

Levels of specific IgA and IgG against MV130 and against each bacteria contained in the mixture (*i.e.*, *S. pneumoniae*, *S. epidermidis*, *S. aureus*, *M. catarrhalis*, *K. pneumoniae*, and *H. influenzae*) were determined in serum samples from blood collected before starting immunotherapy with MV130 and between 4 to 41 months after initiating the treatment. Quantification was performed by ELISA following standard procedures as previously described (26). Once collected, sera samples were stored frozen at −80°C until further processing. Briefly, 96-well non-tissue culture-treated plates were pretreated with poly-L-lysine (Sigma-Aldrich) for 1 h under UV light and coated with each of the heat-inactivated whole cell bacteria or the polybacterial mixture (300 FTU/ml ~ 10^9^ bacteria/ml) overnight at 4°C, and, subsequently, incubated with human serum dilutions for 2 h at room temperature. Specific immunoglobulins were detected with biotin rat anti-human IgA and IgG (both from Sigma-Aldrich). Signal was developed by incubation with streptavidin-horseradish peroxidase (HRP) (Sigma-Aldrich). Peroxidase activity was revealed by the addition of o-phenylenediamine dihydrochloride (Sigma-Aldrich) and the reaction was stopped with HCl 1N. Plates were read on an ELISA reader at 490 nm (Triturus Elisa, Grifols).

### Statistics

Normal distribution was assessed by means of Shapiro-Wilk. Descriptive data are presented as mean ± standard deviation (SD) or median values (range, max - min), according to the normal or non-normal distribution of data, respectively. Data were analyzed by paired Wilcoxon signed-rank test using the Excel spreadsheet (Microsoft, Inc., Redmond,WA, USA) and GraphPad Prism software (GraphPad Software, La Jolla, CA, USA version 8). Differences were considered statistically significant at *p*<0.05.

## Results

### Baseline Characteristics of Patient Population

Fifteen HM patients (aged 42 to 80 years; nine females) that suffered from RRTIs were included in the study. According to the patient’s diagnosis, they were classified as: monoclonal gammopathy of undetermined significance (MGUS) (n = 7, 47%), non-Hodgkin lymphoma (NHL) (n = 6, 40%), chronic lymphocytic leukemia (CLL) (n = 1, 6.5%) and mucosa associated lymphoid tissue lymphoma (MALT) (n = 1, 6.5%). According to the Ann Arbor, Rai and Binet staging of CLL and NHL groups, disease was stage IV (n = 2; 25%), III (n = 3; 37%), II (n = 2; 25%) and one patient with extranodal involvement (13%). In the paraprotein group, four patients were diagnosed of MGUS IgG κappa (n = 2; 29%) and lambda (n = 2; 29%), IgM lambda (n = 2; 29%), and IgA lambda (n = 1; 13%). None of the patients presented abnormal kappa-lambda serum free light chain (FCL) ratio. Regarding comorbidities, six out of eight patients (40%) presented basal bronchiectasis and one patient recurrent herpes infection (**Table 1**).

**Table 1:** Baseline demographic and disease characteristics of patients with B cell hematological malignancies receiving MV130 (N=15).

	n or mean ± SD
**Demographic characteristics**	
Age (years)	66.87 ± 11.42
Age range (years)	42–80
Sex (females)	9
**B cells hematological malignancies classification**	
Monoclonal gammopathy of undetermined significance (IgG Kappa and Lambda; IgM Lambda and IgA Lambda)	7
non-Hodgkin lymphoma (IV, III, and II)	6
chronic lymphocytic leukemia (4Rai, C Binet)	1
Mucosa associated lymphoid tissue lymphoma (I–E)	1
**Comorbidities**	
Bronchiectasis	6
Herpes	1

Excluding the MGUS paraprotein group (n = 7), eight patients showed humoral immune defect: panhypogammaglobulinemia (n = 4; 50%) and dysgammaglobulinemia (n = 4; 50%) (**Supplementary Table 1**). In practice, the determination of vaccine responses is a key criterion to assess immunodeficiency. In this sense, it is important to distinguish between protein and polysaccharide responses, which rely upon different immune pathways. Eighty percent of the patients (n = 13) presented polysaccharide antibody (Ab) deficiency and all of them protein Ab deficiency. Six out of fifteen patients (40%) presented CD4^+^ T-cell lymphocytopenia (between 300 and 500/mm^3^) (**Supplementary Table 1**).

Patients underwent immunization with MV130 daily for 3 months and were followed for a total of 12 months (**Figure 1**). None of the patients were receiving chemotherapy during the immunological evaluation period, last chemotherapy cycle in 2011. Excluding the MGUS paraprotein subgroup (n = 7), three patients (38%) had received one cycle chemotherapy (4–6 weeks), four patients (50%) at least two cycles and one (12%) patient received one cycle chemotherapy and autologous stem cell transplant. During the follow-up period, the HM remained stable in all patients. According to previous hematological history, one patient of the CLL group was splenectomized secondary to refractory autoimmune haemolytic anemia (AHAI). Seven patients out of 15 (47%) required IgRT due to severe immunosuppression 15 ± 4 months post-MV130; six of them on intravenous immunoglobulin (200 mg/kg) and the other on subcutaneous immunoglobulin (300 ml/monthly). Four out of these seven patients (57%) had received more than one cycle of conventional chemotherapy after HM diagnosis and years before MV130, except for one patient who had a diagnosis of MGUS and pulmonary lymphangiomatosis who also required IgRT due to recurrent infections. The remaining untreated patients (non-IgRT) belonged to the MGUS group (n = 6) and NHL group (n = 2). An interesting fact is that one of the NHL patients had indication of IgRT - because of Ig levels below 300 mg/dl and antibody production defect -, and refused to start IgRT, but remained asymptomatic from the infectious perspective after receiving MV130.

### MV130 Decreased the Frequency of Infections in Patients With Hematological Malignancy and Secondary Immunodeficiency

**Figure 2:**
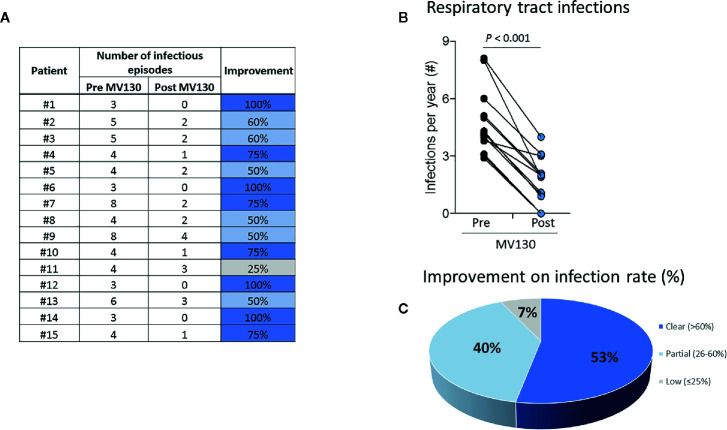
Prophylaxis with MV130 reduces the incidence of respiratory infection rate (%) in patients with B cell hematological malignancies. **(A, B)** Number of respiratory tract infections one year before immunotherapy with MV130 (Pre) and in the 12 months after initiation of immunotherapy (Post) (**A**-tabulated, **B**-graph). **(A, C)** Percentage of improvement (reduction of infections) (**A**-tabulated, **C**-pie chart). Improvement was classified as: clear (>60% reduction of infectious episodes), partial (26-60% reduction of infectious episodes) and low (<25% reduction of infectious episodes). A total of 15 patients were included. Normal distribution was evaluated using Shapiro-Wilk test, and *P* value was calculated comparing infectious episodes pre and post immunotherapy with MV130 using Wilcoxon signed rank test.

Clinical improvement assessed by a decline on the rate of infections was confirmed in all patients undergoing treatment with MV130. The median (range, min-max) of respiratory infections in our cohort of 15 patients dropped from 4.0 (8.0–3.0) before MV130 to 2.0 (4.0–0.0) during the 12 months following the initiation with MV130 treatment (*p*< 0.001) (**Figures 2A, B**).

Follow-up data evidenced a clear improvement in 53% (n = 8) patients, a partial improvement in 40% (n = 6) patients and a low improvement in 7% (n = 1) patients (**Figures 2A, C**). Of note, five out of six (83.3%) patients with basal bronchiectasis experienced a decrease on the number of infectious episodes between 50 and 100%.

One patient with MGUS diagnosis presented low improvement post-MV130 and is highly suspicious of being a primary immunodeficiency (PID) (lung linfangiomiomatosis, bronchiectasis, CD4^+^ T-cell lymphocytopenia), currently awaiting confirmation by genetic results and under IgRT treatment.

Regarding safety, none of the patients reported any adverse reaction related to MV130 treatment, either local at the oral mucosa or systemically, during the 12-month monitoring period.

### MV130 Reduces Antibiotic and Healthcare Resource Consumption

**Figure 3:**
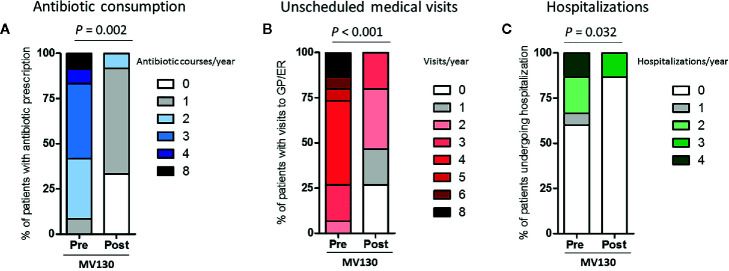
MV130 cuts down on antibiotic and healthcare resource consumption. **(A)** Antibiotic consumption, **(B)** unscheduled medical visits, and **(C)** hospitalizations during the year before (Pre) and after (Post) the initiation of immunotherapy with MV130. Data from n=12 **(A)** n=15 **(B, C)** patients are displayed, Normal distribution was evaluated using Shapiro-Wilk test, and P values were calculated using Wilcoxon signed rank test. GP, general practitioner; ER, emergency room.

Among the secondary outcomes that were evaluated, antibiotic prescriptions, unscheduled GP and Emergency room visits and hospital admissions were included. The significant reduction in respiratory tract infections observed correlated with a decrease in antibiotic consumption. The number of antibiotic cycles (mean [range, min -max]) was reduced from 3.0 (8.0–1.0) 12 months previous to bacterial immunotherapy to 1.0 (2.0–0.0) (*p* = 0.002) during 12 months after initiating the treatment (**Figure 3A**). The number of infectious-related GP and Emergency room visits also declined from 4.0 (8.0–2.0) to 2.0 (3.0–0.0) (*p*<0.001) (**Figure 3B**). Not surprisingly, a significant decrease in hospital admissions was also demonstrated following MV130 treatment (*p* = 0.032) (**Figure 3C**).

### Immunotherapy With MV130 Increases MV130-Specific IgA and IgG Circulating Antibodies

**Figure 4:**
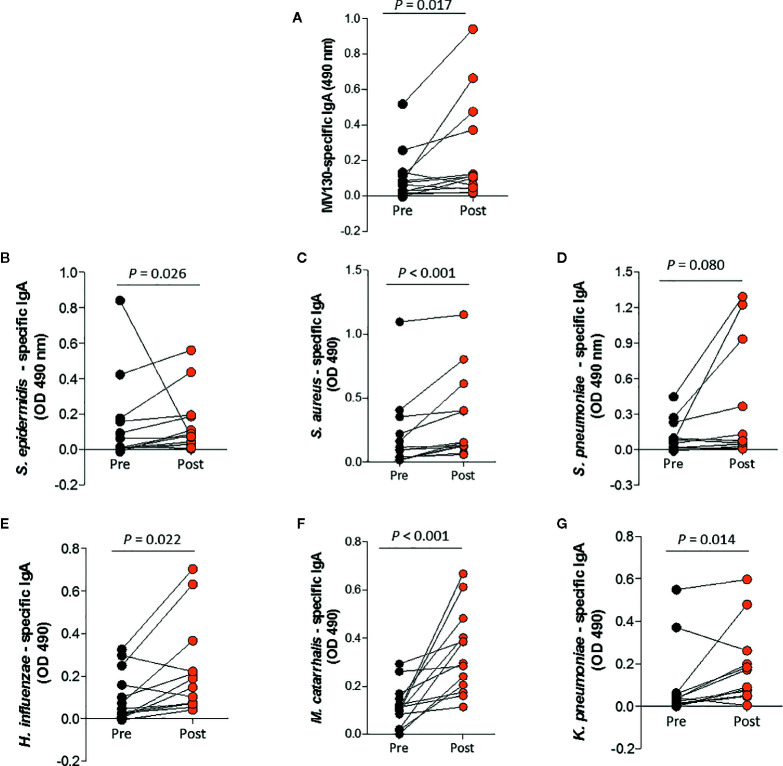
Prophylaxis with MV130 increases serum specific IgA antibody production. Serum IgA specific antibodies against **(A)** MV130 (bacterial mixture), **(B)** S. epidermidis, **(C)** S. aureus, **(D)** S. pneumoniae, **(E)** H. influenzae, **(F)** M. catarrhalis and **(G)** K. pneumoniae were quantified from patients before immunotherapy with MV130 (Pre), and 4 to 41 months after initiation of immunotherapy with MV130 (Post). Levels of antibodies were determined by ELISA. Sera samples from a total of 13 patients were included. Normal distribution was evaluated using Shapiro-Wilk test, and P values were calculated using Wilcoxon signed rank test.

Within the main causes of secondary antibody deficiency (SAD), B cell lymphoproliferative diseases including MGUS, NHL and CLL are very common. In addition, the increased use of B cell targeted therapies where it is used as maintenance and/or combination therapy, results in more patients with SAD (1). Considering that patients with B cell HM have an impaired specific antibody production that is often associated with increased frequency of severe or recurrent infections, we sought to address whether MV130 sublingual immunoprophylaxis could have any impact on the humoral immune response. Median baseline IgG serum level at first documentation was 5.1 g/L and 0.2 g/L for IgA (excluding patients with serum paraprotein). For the MGUS group median baseline IgG was 13.0 g/L and 1.6 g/L for IgA (**Supplementary Table 1**). To this end, blood samples were collected before and after bacterial immunization and sera samples assayed for MV130-specific IgA and IgG titers. We further quantified bacteria-specific IgA and IgG levels for each of the bacterial strains contained in MV130. Higher IgA antibody levels against the whole bacterial preparation and against certain bacterial strains (MV130- (*p* = 0.017), *S. epidermidis*- (*p* = 0.026), *S. aureus*- (*p*<0.001), *H. influenzae*- (*p* = 0.022), *M. catarrhalis*- (*p*<0.001), and *K. pneumoniae*- (*p* = 0.014)) were detected after MV130 treatment (**Figures 4A–G**). A significant increase was also noted for IgG antibodies against MV130- (*p* = 0.039), *S. pneumoniae*- (*p* = 0.013), *S. aureus*- (*p* = 0.033), *H. influenzae*- (*p* = 0.004), and *M. catarrhalis*- (*p* = 0.021) (**Supplementary Figures 1A–G**).

## Discussion

According to health organizations advice for seeking new treatment alternatives against bacterial diseases, and to mitigate many factors to be considered when choosing empirical antibiotic, effective treatment for recurrent infections without side effects and bacterial resistance is necessary (29). Hence, TIbVs have been described useful in preventing recurrent respiratory infections, in both adults and pediatric populations and in the setting of primary immunodeficiency (26, 30, 31). Here, we report the beneficial effects of MV130, a mucosal polybacterial preparation, in a small cohort of HM patients as adjuvant or monotherapy for the prevention of recurrent respiratory infections. Our data show a significant reduction in the frequency rate of RRTIs in the following 12 months of initiating the vaccine. Moreover, three patients that concomitantly suffered from recurrent urinary tract infections did not show any sign of urinary infection during the 12-month follow-up period, highlighting the broad-spectrum of protection conferred by MV130 (15). The clinical benefit of this approach might be probably the highest early at HM diagnosis, when the tumor burden is low and before tumor progression has resulted in profound immune exhaustion (12). Mucosal TIbVs induce an immune response that more closely resembles natural immunity than the response elicited by injectable vaccines. Thus, mucosal routes of immunization are better alternatives than parenteral immunization to enhance immunity in tissues such as lung mucosa (32). Our results show clinical improvement of respiratory tract infections after MV130 immunization on five out of six patients with basal bronchiectasis. This fact might be supported by previous studies that demonstrate that Th17 T-cell response enhanced endobronchial clearance in COPD patients following oral immunization (33). In this line, among the mechanisms of actions previously described for MV130, it has been demonstrated that it enhances both specific and non-specific T cell responses *in vitro* and *in vivo*, including Th1 and Th17 immune responses (23).

Previous clinical observations have also suggested an antiviral effect for MV130 related to the existence of helper CD4^+^ T-cells activity associated with effective immune responses in long-term therapeutic immunizations (19). Prevalence of viral infections in HM patients is high and can deteriorate the patient’s condition and disrupt the treatment process. For that reason, the prevention and treatment of respiratory viral infections in cancer patients are of great importance. In this line, the efficacy of MV130 was highlighted in a clinical trial in children with recurrent wheezing, a condition triggered in most cases by viral infections (respiratory syncytial virus and rhinovirus primarily, but also by others such as flu virus or coronavirus). In that trial, a significant decrease in the number and duration of wheezing attacks, together with a reduction in symptoms and medication score was assessed in MV130 group over placebo (22). It has been postulated that a trained immunity-mediated mechanism might be underlying the protection conferred by MV130 in this clinical trial. In our cohort, a patient who presented with recurrent herpes simplex infections (7 episodes prior to MV130), did not show any recurrence of herpes during the 12-months follow-up period following sublingual vaccination, which suggests that the bacterial preparation may have induced an immune stimulating effect that enhanced the ongoing cellular immune response to viral antigens (19). Indeed, in a former report where 17 adult patients with RRTIs and suspected primary immunodeficiency were treated with MV130 for 6 months, one of those subjects presented 12 episodes of labial and nasal herpes virus prior to MV130 mucosal immunotherapy, this number decreased to three episodes during the year following the beginning of the immunotherapy (19).

Using an experimental approach, it has been shown that mucosal exposure to bacterial extracts provides protection against respiratory viral infection (influenza), enhancing mucosal and systemic antibody production (34). HM patients often present very low antibody production associated with an increased frequency of severe or recurrent infection. Despite the inherent antibody defect observed in our cohort of HM patients, a significant MV130- specific IgA and IgG serum titers, together with and increased in pathogen-specific IgA and IgG levels of bacterial strains contained in the bacterial preparation, were detected following MV130 mucosal immunotherapy. These results highlight the ability of sublingual MV130 administration to induce an immunogenic response against MV130-containing bacteria in these low-responder patients. Of note, these bacterial strains are among the most common pathogens of the respiratory tract. Therefore, supporting a specific as well as a non-specific protective role of MV130. These results are one of the main strengths of this study, we believe it may be somehow underlying the mechanism of protection conferred by MV130 reducing the recurrence of infection in these patients. Furthermore, these data support a recently published report where patients with common variable immunodeficiency that received MV130 immunotherapy declined their rate of respiratory infections in parallel with an increase in MV130-specific IgA serum antibodies (26). The potential of MV130 to offer certain protection to infection by the recent SARS-CoV-2 virus in this population would also be interesting to explore.

Despite we are gaining insight into the mechanism of action of mucosal bacterial immunotherapy, it is not yet fully understood. Recently, trained immunity-based vaccines have been described as anti-infectious vaccines composed of whole microorganisms or derived products that induce trained immunity and confer heterologous protection (15). Several effects of TIbVs have been described, including different molecular mechanisms such as a metabolic switch from oxidative phosphorylation to aerobic glycolysis, epigenetic reprogramming of innate immune cells and enhancement of T-cell adaptive responses (35). Long-lasting epigenetic changes in innate immune cells may be effective not only against the specific pathogens targeted by the vaccine but also to improve immune responses to possible bystander pathogens, acquiring a higher resistance to a second infection (cross-protection) for a relatively prolonged time period (11). In addition, clinical studies have pointed out trained immunity as a novel approach to enhance responses against infection and vaccination in the elderly and other vulnerable populations when innate immune cells display functional defects, such as decrease in the phagocytosis and cytokine production, and adaptive immune cells exhibit defective antibody production and/or a decline in naïve cell population (36). Certain bacterial preparations that have previously demonstrated clinical benefit reducing recurrent infections such as MV130, have been postulated as TIbVs for their ability to promote host resistance against a wide spectrum of pathogens (19, 21, 25, 26). In this regard, we have not evaluated the ability of MV130 to induce trained immunity in the cohort of patients studied, but we have rather assessed an increase in antibody production. Whether trained immunity, antibody responses or both mechanisms are underlying the protection conferred by MV130 in this setting needs further evaluation. The MV130 vaccine has been used for more than 10 years in Spain financed by the National Security Health System, and there is a large amount of available data on its safety and in different populations (19, 21, 22, 26). Although our study is limited by the small cohort of our HM patients, the retrospective design and the absence of a control group, our results are very encouraging as preliminary data. However, future properly designed clinical trials including sample size calculation and exclusion of confusion factors, such as IgRT usage, are needed to validate the utility of MV130 in SID diagnosis.

Despite the fact that the economic impact of MV130 immunoprophylaxis has not been addressed in the present study, we speculate that the significant drop in antibiotic consumption, infectious-related GP/ER visits and hospital admission might reflect a concomitant reduction in medical (healthcare resource expenses) and non-medical direct and indirect costs. This has been proven in previous studies carried out with MV130 where direct costs such as surgery, cycles of antibiotics, hospitalization and indirect costs such as work/school absenteeism, among others, have been assessed (21, 26). Therefore, providing an additional advantage for this interventional approach.

## Conclusions and Future Perspectives

This study is a compilation of our clinical practice experience and to our knowledge, this is the first study to report clinical data with TIbV in a group of HM patients.

Furthermore, since the purpose of these vaccines is to confer broad-spectrum protection, the primary efficacy endpoints must be designed in relation to clinical response. However, in the absence of standardized protocols, individual decision should be made with a balance of risks and benefits. TIbV may add clinical benefit to current treatments and it is important to note that it is a non-exclusive prophylactic strategy in HM with recurrent infections.

To date, no clear guidelines or data exist that compares the use of TIbV *versus* antibiotic prophylaxis in the prevention of recurrent infections in HM patients. To validate these data, further properly designed clinical trials are warranted to determine the clinical impact of TbIV in HM patients, also helping to clarify several open questions: 1) How long does antibiotic prophylaxis should be maintained in HM patients? 2) Are there any differences in the clinical outcome after prophylactic treatment discontinuation *versus* patients who have received TIbV? 3) Would it be advisable to give more than one cycle of TIbV in patients where infectious episodes recur or before clinical appears? 4) May polyvalent sublingual vaccine have greater benefit in patients with IgA deficiency? And finally, 5) would personalized TIbV be an alternative in patients with viral infections, bronchiectasis, or *Pseudomonas* infection?

## Data Availability Statement

The raw data supporting the conclusions of this article will be made available by the authors, without undue reservation.

## Ethics Statement

The studies involving human participants were reviewed and approved by Hospital Clínico San Carlos, Madrid (Spain) institutional research ethics committee. Written informed consent for participation was not required for this study in accordance with the national legislation and institutional requirements, since data were collected conforming medical records as per routine clinical practice.

## Author Contributions

JO-G and SS-R contributed to conception and design of the study. AR and CD-R have contributed to specific immunological studies. GC, ER, and KG-H contributed to the database. JO-G wrote the first draft of the manuscript. LC contributed to the writing of the manuscript and statistical analysis of data and figures. SS-R wrote sections of the manuscript and reviewed the manuscript. CD-R contributed to the measurement of specific antibodies and writing of the manuscript. All authors contributed to the article and approved the submitted version.

## Conflict of Interest

LC and CD-R belong to the Immunotek R+D Department.

The remaining authors declare that the research was conducted in the absence of any commercial or financial relationships that could be construed as a potential conflict of interest.

## Abbreviations

CLL, chronic lymphocytic leukemia; COPD, chronic obstructive pulmonary disease; HM, hematological malignancy; IgRT, inmmunoglobulin replacement therapy; MALT, mucosa-associated lymphoid tissue lymphoma; MGUS, monoclonal gammopathy of undetermined significance; NHL, non-Hodgkin lymphoma; RRTIs, recurrent respiratory tract infections; TIbV, trained immunity-based vaccines; URTI, urinary tract infections.
