# Supplementary figures and images for: Trained Immunity-Based Vaccine in B Cell Hematological Malignancies With Recurrent Infections: A New Therapeutic Approach
Source: Front Immunol. 2021 Feb 12;11:611566. doi: 10.3389/fimmu.2020.611566 (PMC7928395; doi:10.3389/fimmu.2020.611566)

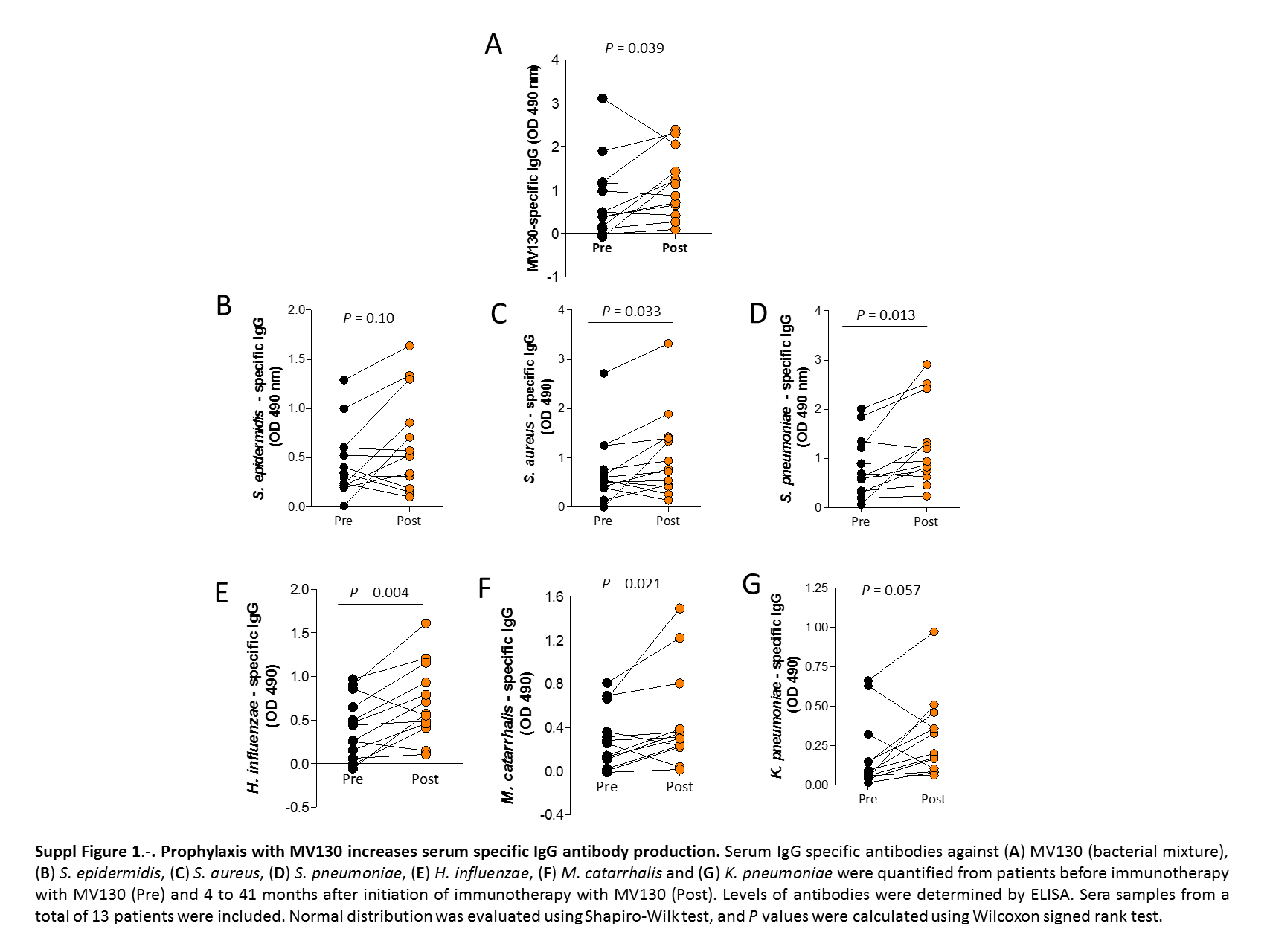

Supplement: Supplementary file 1 [file Image_1.tif]
