# Supplementary material for: Trained Immunity-Based Vaccine in B Cell Hematological Malignancies With Recurrent Infections: A New Therapeutic Approach
Source: Front Immunol. 2021 Feb 12;11:611566. doi: 10.3389/fimmu.2020.611566 (PMC7928395; doi:10.3389/fimmu.2020.611566)
Supplement: Supplementary file 2 [file Table_1.pdf]

Suppl. Table 1. Immunological assessment per group prior to MV130 vaccination

	**MGUS**<br>**No: 7**	**NHL**<br>**No: 6**
**IgG (mg/dL)**	1,302±722<br>1,062(1590)	484±194<br>431 (274)
**IgA (mg/dL)**	164±131<br>82 (279)	13±12<br>8 (24)
**IgM (mg/dL)**	871± 1720<br>110 (419)	45±34<br>49 (75)
**IgG1 (mg/dL)**	653±335<br>476 (719)	315±231<br>285 (422)
**IgG2 (mg/dL)**	653±335<br>476 (719)	96±60<br>90 (124)
**Lymphocytes count**	1,442±509<br>1,200 (900)	2,066±869<br>1,850 (1,275)
**%B lymphocytes**	13±16<br>7 (15)	9±5<br>10 (10)
**B cell lymphocytes (/µL)**	129±62<br>136 (106)	146±66<br>168 (127)
**%$CD4^+$ T-lymphocytes**	37±15<br>40 (29)	30±7<br>28 (8)
**$CD4^+$ T-lymphocytes (/µL)**	649±405<br>587 (623)	629±264<br>526 (537)
**%NK cells**	14±6<br>13 (12)	16±9<br>12 (20)
**NK cells (/µL)**	295±139<br>273 (294)	265±206<br>190 (431)

Data are presented as mean (Standard Deviation; SD); median (Interquantilic Range; IQR).
Data of CLL and MALT patients are not presented (n=2).
